# Effects of Different Additives on the Quality of Rice Straw Haylage, Ruminal Fermentation Parameters and Methane Production in Hu Sheep

**DOI:** 10.3390/ani15243573

**Published:** 2025-12-12

**Authors:** Jun Deng, Lin Wang, Chunbin Zheng, Zihan Gao, Zhongju Li, Rui Su, Weihao Chen, Xiaoyang Lv, Wei Sun

**Affiliations:** 1 College of Animal Science and Technology, Yangzhou University, Yangzhou 225009, China; eric.deng@wuitu.com (J.D.); lynn1215@yzu.edu.cn (L.W.); 17332365645@163.com (Z.G.); 18552133709@163.com (W.C.);; 2 Changzhou Wuitu Smart Technology Co., Ltd., Changzhou 213000, China; 3 Joint International Research Laboratory of Agriculture and Agri-Product Safety, Institute of Agricultural Science and Technology Development, Yangzhou 225009, China; 4 Suzhou Breeding Sheep Farm Co., Ltd., Suzhou 215228, China; surui99114074@163.com

**Keywords:** rice straw, methane, Hu sheep

## Abstract

In this study, eight treatment groups were set up with three replicates in each group to investigate the effects of Bacillus, Lactobacillus, and molasses on the chemical composition and fermentation quality of fermented rice straw. Furthermore, an animal experiment was conducted to determine the nutrition apparent digestibility, ruminal fermentation characteristics, and methane emission in Hu sheep. The results showed that the dry matter (DM) and crude protein (CP) content in the group treated with *Bacillus megaterium* and *Lactobacillus acidophilus* (BMLB) was significantly higher than that in the group with no additive (CK) (*p* < 0.05). Compared with the CK group, all treatments with single or composite addition of Bacillus significantly reduced the content of neutral detergent fiber (NDF) and acid detergent fiber (ADF) (*p* < 0.05). The content of acetate, propionate, and NH_3_-N in the BMLB group were significantly lower than those in the CK group (*p* < 0.05). In addition, the Hu sheep in the BMLB group showed a significant reduction in daily methane emission per unit of metabolic body weight. In conclusion, the BMLB treatment significantly improved the nutritional value, fermentation quality, ruminal fermentation, and methane emission of rice straw haylage in Hu sheep.

## Simple Summary

Rice straw, as a type of agricultural waste, is stored in abundance in China. It is an excellent choice to alleviate the shortage of high-quality roughage and can be used as roughage to feed ruminants. However, its high content of lignin and cellulose results in low palatability and digestibility. The first objective of this study was to investigate the effects of different additives on the chemical composition and fermentation quality of rice straw haylage. Moreover, due to the different behavior of the digestive system in vivo compared with in vitro, an animal trial was conducted to determine the effect of rice straw haylage on ruminal fermentation characteristics and methane emission in Hu sheep. This study suggests that rice straw haylage treated with BMLB can improve fermentation quality and decrease the methane emission per unit of metabolic body weight.

## 1. Introduction

Rice straw is a major agricultural by-product in China, with an annual production of approximately 200 million tons. However, its utilization rate remains below 60%, leading to potential environmental pollution and resource waste [1,2]. The rapid development of China’s animal husbandry industry has increased the demand for roughage. The rational use of straw-based feed represents an effective approach to mitigating environmental impacts and enhancing feed supply. Rice straw is rich in fiber, an essential component of ruminant diets, which can be broken down by rumen microbes to provide energy, support rumen health, and maintain digestive function [3,4,5,6]. Nevertheless, due to its high lignin content, low nutrient digestibility, and poor palatability, it is difficult to meet the nutritional requirements of livestock production. Therefore, improving the quality of rice straw as feed is crucial for promoting its utilization in ruminant production.

Although ensiling is an effective method to enhance the nutritional value and palatability of rice straw, its low content of water-soluble carbohydrates (WSC) and limited epiphytic lactic acid bacteria often restrict its fermentation quality [7,8,9]. Studies have indicated that appropriate additives, such as fermentation stimulants (e.g., lactic acid bacteria), substances that inhibit undesirable fermentation (e.g., propionic acid, formic acid), nutritional additives (e.g., molasses, urea), and absorbents (e.g., straw, bran), can significantly enhance both the nutrient content and fermentation quality of rice straw silage [10,11,12,13,14,15,16]. For instance, it was reported that adding molasses promotes a dominant lactic acid fermentation pathway, stimulates lactic acid production during ensiling, lowers the pH, and reduces organic matter loss [17]. This approach is particularly beneficial for feedstocks with low inherent WSC. Similarly, it was also demonstrated that lactic acid bacteria utilize fermentable carbohydrates to produce abundant lactic acid, leading to a rapid decline in pH and suppression of spoilage microorganism growth, thereby minimizing nutritional losses associated with aerobic degradation [18,19].

Bacillus is a Gram-positive, spore-forming bacterium with cellulose-degrading capabilities [20,21,22]. In our previous studies, *B. megaterium* was shown not only to promote the digestion of nutrients such as protein, regulate intestinal pH, enhance antioxidant capacity, and improve product quality, but also to significantly increase the digestibility of neutral detergent fiber in rumen and optimize ruminal fermentation patterns, and thereby improve feed utilization efficiency [23,24]. These findings suggest that Bacillus might be able to degrade the fiber. Several studies have demonstrated the positive effects of different Bacillus strain-based additives on fermented forage quality, such as alfalfa, corn, oats, and so on [25,26]. Therefore, the objective of this study was to evaluate the effects of *B. megaterium* with different additives on the fermentation quality of rice straw feed. Furthermore, we carried out an animal feeding trial to examine the effects of *B. megaterium*-treated rice straw haylage on nutrient digestibility, ruminal fermentation characteristics, and methane emissions in Hu sheep.

## 2. Materials and Methods

### 2.1. Experimental Materials

The rice straw utilized in this experiment was collected from the experimental field at Yangzhou University (harvested in October), with a stubble height of 7 cm. It was chopped into 2–3 cm lengths for use. The nutritional composition of the rice straw was analytically determined. On a dry matter (DM) basis, the chemical compositions of the rice straw were as follows: DM 66%, crude protein (CP) 4.0%, ash 20.5%, neutral detergent fiber (NDF) 71%, and acid detergent fiber (ADF) 54%.

Bacillus (*B. megaterium*, 4 × 10^10^ CFU/g) was obtained from the Animal Nutrition and Feed Engineering Technology Research Center of Yangzhou University. The Bacillus strain, *B. megaterium* 1259, was typically facultative anaerobes, and was isolated from chicken manure and deposited in the China General Microbiological Culture Collection Center (CGMCC No 1259). Lactic acid bacteria (*L. acidophilus*, 1 × 10^10^ CFU/g) were purchased from the ZhongHuiLeNong company (Zhengzhou, China), and molasses was purchased from the Molasses company (Guigang, Guangxi, China).

### 2.2. Haylage Preparation

Ten grams of *B. megaterium* powder (4 × 10^10^ CFU/g) was dissolved in 1 L of deionized water to obtain a dilution with a concentration of 4 × 10^8^ CFU/mL. Similarly, 4 g of *L. acidophilus* powder (1 × 10^10^ CFU/g) was dissolved in 1 L of deionized water to prepare a dilution containing 4 × 10^7^ CFU/mL. In addition, 10 mL of molasses was dissolved in 490 mL of deionized water to prepare a 2% molasses solution. The chopped rice straw was then mixed with the additives and divided equally into eight treatment groups as follows. (1) CK: no additive; (2) BM: 5 mL of a *B. megaterium* dilution (4 × 10^8^ CFU/mL) plus 95 mL of deionized water; (3) LB: 5 mL of a *L. acidophilus* dilution (4 × 10^7^ CFU/mL) plus 95 mL of deionized water; (4) BMLB: 5 mL of the *B. megaterium* dilution, 5 mL of the *L. acidophilus* dilution, and 90 mL of deionized water; (5) M: 5 mL of a 2% molasses solution plus 95 mL of deionized water; (6) MBM: 5 mL of the 2% molasses solution, 5 mL of the *B. megaterium* dilution, and 85 mL of deionized water; (7) MLB: 5 mL of the 2% molasses solution, 5 mL of the *L. acidophilus* dilution, and 90 mL of deionized water; and (8) MBMLB: 5 mL of the 2% molasses solution, 5 mL of the *B. megaterium* dilution, 5 mL of the *L. acidophilus* dilution, and 85 mL of deionized water.

A total of 200 g of each mixed sample was packed into polyethylene bags (30 cm × 20 cm) and vacuum-sealed. Five bags were prepared for each treatment. All bags were ensiled at ambient temperature. All bags from each group were opened on day 30 for further analysis.

### 2.3. Chemical Composition and Fermentation Characteristics of Ensiling

The polyethylene bags were aseptically opened, and ninety-gram samples were collected for drying and grinding through a 40-mesh sieve. DM content was determined by oven-drying at 65 °C for 48 h. CP was estimated following the methods established by the Association of Official Analytical Chemists [27]. NDF and ADF were analyzed according to Van Soest et al. (1991) [28].

Another twenty grams of the samples were blended with 45 mL of deionized water and extracted in a refrigerator at 4 °C for 24 h. The mixture was then homogenized using a juice extractor and filtered through four layers of cheesecloth. The extract was used to determine pH, lactic acid, acetic acid, propionic acid, butyric acid, and NH_3_-N content. The pH was measured directly with a pH meter. NH_3_-N content was assessed by the phenol-sodium hypochlorite colorimetric method [29], and organic acid composition was analyzed using gas chromatography [30]. For microbial analysis, a 20 g haylage sample was homogenized with 180 mL of sterile saline and serially diluted. The counts of lactic acid bacteria, *E. coli*, and Bacillus bacteria were enumerated using the agar plate method.

### 2.4. Animal Experiment

An animal experiment was conducted at the Suzhou Sheep Farm (Suzhou, China). All experimental procedures involving animals were carried out in accordance with laws and regulations approved by the Animal Welfare Office of Yangzhou University (No. 202410750). A total of twenty-four male Hu sheep with a mean age of three months and similar body weights were selected and divided into two groups using a randomized block design. The experiment included two treatments: for the control group (CK), Hu sheep with similar body weights (25.2 ± 1.07 kg) received fermented rice straw without any additives, and for the treatment group (BMLB), Hu sheep with similar body weights (24.9 ± 1.12 kg) received fermented rice straw supplemented with a combination of *B. megaterium* and *L. acidophilus*. The sheep were fed the experimental diets at 2% of their body weight (BW) on a DM basis, provided in two equal portions daily at 08:30 h and 17:30 h. The animals had free access to water and mineral blocks. The adaption period was one week, and the experiment diets were fed over a period of four weeks.

CH_4_ emissions were measured by using an open-circuit respiratory calorimetry system, which was equipped with a gas chromatograph (HKYC Technology Co., Ltd., Beijing, China). The system was connected to six respiratory chambers, allowing for simultaneous measurements from six sheep. Each respiratory chamber contained a feed trough and a water trough, providing ad libitum access to the experimental diet and water. After feeding sheep with the experimental diet for four weeks, the sheep were moved into the respiratory chambers. Following a 1-day adaptation period in the chambers, CH_4_ emissions were recorded over two consecutive days. The daily CH_4_ emission per sheep was calculated by summing the results from all measurement cycles.

During the period for measuring CH_4_ emissions in chambers, all feces and urine from the Hu sheep were collected every morning before feeding. Feces collected over three days from each Hu sheep were pooled, and 50 g of the mixed sample was ground using a Wiley mill (model 1188Y, Thomas Willey, NJ, USA) to pass through a 1 mm sieve, then stored for further analysis. Urine was collected into plastic containers pre-filled with 100 mL of 10% H_2_SO_4_ to stabilize nitrogenous compounds. The total volume of urine was recorded. The urine was then mixed thoroughly and filtered through four layers of cheesecloth. A 10% aliquot of the total daily urine output was collected. Rumen fluid samples were collected via an oro-ruminal stomach tube before feeding on the final morning of the experimental period in the chamber. The initial aliquot was discarded to avoid saliva contamination, and the subsequent sample was collected into pre-warmed, anaerobic containers for subsequent analysis. The fluid was filtered through four layers of gauze and pH was immediately measured using a glass electrode pH meter. It was then centrifuged at 15,000× *g* for 15 min, and the supernatant was stored at −20 °C for VFA analysis [30]. Feed digestibility was calculated according to the method described by Zewdie (2019) [31].

### 2.5. Statistical Analysis

All data were analyzed using the ANOVA procedure in SAS 9.3 software (SAS Institute Inc., Cary, NC, USA). Significant differences among means were further evaluated by Duncan’s multiple range test, and differences at *p* < 0.05 were considered statistically significant.

## 3. Results

### 3.1. Effects of Different Additives on the Chemical Composition of Rice Straw Haylage

As shown in Table 1, the DM content in all the treated groups was significantly higher than that in the CK groups (*p* < 0.05). The CP content in the MBMLB, MLB, MBM, BMLB, LB, and BM groups showed a significant increase compared with the CK group (*p* < 0.05). The NDF content in the BMLB, BM, and M groups was significantly lower than that in the CK group. In addition, the BMLB group had significantly lower ADF content compared to the other groups.

### 3.2. Effects of Different Additives on Fermentation Quality of Rice Straw Haylage

As shown in Table 2, the pH in the BMLB group was significantly lower than those in the CK, BM, and M treatment groups (*p* < 0.05). The NH_3_-N content was significantly reduced in the BMLB, MLB, and MBMLB groups compared with the CK group (*p* < 0.05). Acetic acid content in the CK and MBM groups was significantly higher than in the other treatments (*p* < 0.05), with the BMLB group exhibiting the lowest value significantly. In addition, lactic acid content was highest in the BMLB group and significantly higher than those in the CK, BM, M, and MBM groups (*p* < 0.05).

### 3.3. Effects of Different Additives on Lactic Acid Bacteria, E. coli, and Bacillus Counts in Rice Straw Haylage

In Table 3, all the other treatments significantly increased the counts of lactic acid bacteria, compared with the control group (CK). Regarding *E. coli*, the counts in BMLB, M, MLB, and MBMLB treatments were significantly decreased than that in CK group (*p* < 0.05). By comparing to the CK, the BMLB group had significantly higher Bacillus count.

### 3.4. Effects of BMLB Supplemented Rice Straw Haylage on Nutrient Apparent Digestibility of Rice Straw Haylage in Hu Sheep

The effects of rice straw haylage supplemented with BMLB on nutrient apparent digestibility in Hu sheep are presented in Table 4. The apparent digestibilities of CP and NDF in the BMLB treatment group were significantly higher than those in the control group. No significant difference was observed between CK and BMLB in DM and ADF digestibilities.

### 3.5. Effects of BMLB Treatment on Ruminal Fermentation Characteristics in Hu Sheep Fed with Rice Straw Haylage

As shown in Table 5, rice straw haylage treated with BMLB significantly affected the rumen fermentation characteristics of Hu sheep. The results showed that the ruminal propionate content was significantly higher in the BMLB treatment group, while the acetate-to-propionate ratio was significantly lower compared to the CK group. In addition, ruminal ammonia content was also significantly reduced in the BMLB treatment group.

### 3.6. Effects of BMLB Treatment on Methane Emissions from Hu Sheep Fed Rice Straw Haylage

As shown in Table 6, although daily CH_4_ emission did not differ significantly between the CK group and BMLB treatment group, methane emission per unit of metabolic body weight per day was significantly reduced in the BMLB treatment group.

## 4. Discussion

### 4.1. Effects of Different Additives on Nutritional Components and Fermentation Quality of Rice Straw Haylage

The addition of *B. megaterium*, *L. acidophilus*, and molasses during the ensiling process improved the nutritional composition and fermentation quality of rice straw haylage. In this study, the nutritional components of rice straw haylage were evaluated for individual or composite additions of the above additives. The highest DM content was observed in the BMLB group, which indicated that adding *B. megaterium* and *L. acidophilus* could reduce dry matter losses [32]. Although the CK group also retained relatively high DM content, it exhibited inferior haylage quality compared with the other groups, which can be attributed to limited fermentation activity. In contrast, the lower DM content in the BM, MBM, MLB, and MBMLB groups indicated enhanced fermentation, in which part of the substrate was converted into volatile products such as VFAs and CO_2_ [33,34].

Compared with the CK group, the BMLB group exhibited higher CP content and lower NDF and ADF content, suggesting that the synergistic interaction between *B. megaterium* and *L. acidophilus* might stimulate the active breakdown of cellulose, yielding more soluble sugars. This process suppressed the growth of undesirable microorganisms, encouraged the proliferation of beneficial microbes, resulted in the accumulation of protein-rich products, and limited the degradation of protein and amino acids [35,36,37]. NDF and ADF are key indicators of fiber quality and palatability; lower fiber levels can enhance palatability, intake, and digestibility [31,32,33]. In this study, the BMLB group showed the lowest NDF and ADF content, which might be because the combined application of *B. megaterium* and *L. acidophilus* could enhance enzyme secretion, leading to the effective degradation of structural carbohydrates in the rice straw, which was explained in other studies [38,39,40]. In addition, we chose 30 days as the opening time because in our preliminary experiment, we investigated the effect of different bag-opening days (1 day, 7 days, 15 days, 30 days, and 45 days) on the quality of rice straw haylage. The results showed that no significant difference was observed between 30 days and 45 days among the treatment.

An appropriate pH helps stabilize the ruminal environment, promotes microbial metabolism and crude fiber degradation and digestion, and enhances fermentation efficiency [37]. The ideal pH range for haylage varies depending on its dry matter content. For grass-based haylage with a dry matter content between 40 and 55%, the pH range is from 4.5 to 5.5 [41]. High-quality forage grasses, such as ryegrass and fescue, which are inherently high in water-soluble carbohydrates and low in lignin content, can promote effective lactic acid fermentation under appropriate dry matter conditions. Conversely, in this study, the inferior quality of the rice straw, with low sugar content and high linin content, resulted in inferior fermentation quality and consequently a higher pH value. According to the fermentation quality results, all additive-treated groups showed decreased pH, increased lactic acid content, reduced NH_3_-N concentration, and lower butyric acid content compared with the control group. In this study, the NH_3_-N concentration in all groups fell within the normal range (0.8–56.1 mg·dL^−1^) [42], indicating that the various additive treatments effectively suppressed protein hydrolysis, which can be attributed to the inhibition of proteolytic enzyme activity under the acidic environment produced during haylage fermentation, leading to reduced NH_3_-N levels. Moreover, the combined application of additives outperformed individual additions, with the composite of Bacillus and lactic acid bacteria showing the most pronounced effects. This indicates that rice straw haylage treated with *B. megaterium* is more effective in improving the fermentation quality of feed, thereby supplying more energy to the animal. Previous studies have shown that incorporating additives such as fermentation promoters, nutritional supplements, and microbial inoculants during the ensiling of rice straw can optimize fermentation quality by modulating metabolic pathways. In particular, the synergistic effect of suitable concentrations of these additives can significantly stimulate the growth of beneficial microorganisms such as lactic acid bacteria, enabling them to produce large amounts of lactic acid during fermentation. Lactic acid accumulation effectively lowers the pH during ensiling. Furthermore, the resulting acidic environment improves microbial utilization of NH_3_-N, promotes microbial protein synthesis, reduces NH_3_-N concentration during fermentation, and ultimately enhances the fermentation quality of rice straw haylage.

It should be noted that we confirmed that the combined addition of *B. megaterium* and *L. acidophilus* (the BMLB group) was the optimal treatment for enhancing the nutritional composition of the rice straw haylage according to results from experiment 1 in our study. Consequently, the BMLB group was selected for the subsequent animal experiment. However, it has been widely reported that rice straw itself has a low intrinsic nutrient content, necessitating the addition of external carbon sources such as molasses during fermentation. In this study, the MBMLB group did not differ significantly from the BMLB group in some nutritional and fermentation parameters. Furthermore, the high acetate-to-propionate ratio was observed in the ruminal fluid of the animal trial, which suggested that feeding animals solely with rice straw silage may not improve rumen fermentation characteristics. Therefore, further research utilizing a total mixed ration (TMR) with rice straw haylage is necessary to meet the nutritional requirements for growth in Hu sheep, and to evaluate the effects of rice straw silage on the growth performance and rumen fermentation characteristics of Hu sheep.

### 4.2. Effects of BMLB Treatment on Nutrient Digestibility and Ruminal Characteristics of Rice Straw Haylage in Hu Sheep

In this study, the digestibilities of CP and NDF were significantly improved compared with the CK group, indicating that the composite addition of *B. megaterium* and *L. acidophilus* might enhance the nutritional value of rice straw and promote its utilization by ruminants [26,43,44,45,46,47]. However, further analysis is necessary to confirm whether the relevant enzymes’ activity is improved.

Ruminal pH is a critical factor for the growth and reproduction of rumen microorganisms, and maintaining a stable pH within the optimal range of 5.6 to 7.5 is essential [47]. In this experiment, the pH values of rumen fluid in all groups remained within the normal range [48], reflecting a stable rumen environment favorable for microbial growth and metabolism.

Ruminants obtain 70% to 80% of their energy from VFAs produced by rumen microbial metabolism [49]. The main VFAs, acetate, propionate, and butyrate, collectively account for approximately 95% of the total. VFAs not only serve as important indicators of feed utilization but also help regulate gastrointestinal hormone secretion and the growth and proliferation of intestinal epithelial cells. Specifically, acetate contributes to milk fat synthesis, while propionate can be converted into glucose, supporting animal fattening. VFA production is influenced by multiple factors, including dietary composition, additives, and the internal rumen environment, with feed composition being the most critical. Optimizing dietary formulations can significantly enhance VFA yield. As a major energy source for ruminants, VFAs participate in key metabolic processes, and their production and regulation are vital for energy balance and overall growth [50,51]. In this study, acetate and propionate levels were higher in the BMLB group than in the other groups, suggesting that the combined addition of Bacillus and lactic acid bacteria improves haylage quality.

The ammonia concentration of rumen fluid remained within the normal range, which was consistent with the findings of Owen and Zinn, who indicated that microbial growth requires ammonia levels between 0.35 and 29 mg/100 mL of rumen fluid [52]. The significantly lower NH_3_-N content in the BMLB group indicates that rumen microbiota efficiently utilized ammonia, converting it into high-quality microbial protein and thereby improving nitrogen utilization efficiency.

### 4.3. Effects of BMLB Treatment on Methane Emissions of Rice Straw Haylage in Hu Sheep

Methane, as a product of ruminal fermentation, is one of the contributors to enhanced global warming [53]. Therefore, reducing methane emissions from ruminants can promote ecological animal husbandry. The reduction in methane emissions in ruminants can be achieved primarily by decreasing hydrogen production, reducing the population of methanogens, and improving rumen fermentation performance, all of which can effectively lower methane production [54,55]. On the other hand, agricultural crop straw is an abundant roughage resource for ruminants; however, its nutritional value is limited and it can lead to elevated methane emissions. Pretreatment via microbial fermentation has been demonstrated as an effective strategy to enhance its nutritive value, improve utilization efficiency, and concurrently reduce ruminal methane production. In our study, compared with the control group, the Hu sheep in the BMLB group showed a significant reduction in daily methane emission per unit of metabolic body weight, which may be attributed to the improved rumen fermentation performance promoted by the fermented rice straw haylage, leading to enhanced propionate synthesis and, consequently, reduced methane emission [7,56]. However, no significant difference in methane production was observed between the BMLB group and the CK group, which warrants further validation through the next animal experiment. It should be noted that the effects of BMLB treatment on the methane emissions of rice straw haylage in Hu sheep were determined in this study; however, since the sheep in this experiment were fed with rice straw haylage for five weeks, the results of methane production could not reflect for the sheep under adequate nutrition conditions. Therefore, further experiment is necessary to measure the methane emission from sheep fed with rice straw haylage in formulated nutrition.

## 5. Conclusions

In summary, the BMLB treatment could significantly improve the nutritional value and fermentation quality of rice straw haylage compared to the CK. Moreover, feeding with BMLB-treated rice straw haylage could increase the digestibilities of CP and NDF, and reduced the daily methane emission per unit of metabolic body weight in Hu sheep. Further research is necessary to evaluate the effects of BMLB in a TMR diet for ruminants.

## Figures and Tables

**Table 1:** Different additives on the chemical composition of rice straw haylage.

Items	Treatments								SEM	*p*-Value
	CK	BM	LB	BMLB	M	MBM	MLB	MBMLB		
DM (%)	53.01 ^c^	63.89 ^ab^	63.33 ^ab^	65.51 ^a^	63.92 ^ab^	61.09 ^ab^	62.84 ^ab^	64.73 ^ab^	1.422	0.048
CP (%DM)	3.64 ^d^	3.88 ^bc^	4.03 ^ab^	4.05 ^ab^	3.81 ^cd^	3.95 ^bc^	3.89 ^bc^	4.16 ^a^	0.841	0.047
NDF (%DM)	72.37 ^a^	66.97 ^b^	70.07 ^ab^	67.43 ^b^	67.00 ^b^	69.23 ^ab^	69.87 ^ab^	69.50 ^ab^	0.154	<0.001
ADF (%DM)	58.57 ^a^	58.19 ^a^	58.55 ^a^	51.17 ^b^	57.58 ^a^	59.28 ^a^	57.78 ^a^	55.10 ^ab^	0.053	0.024
Ash (%DM))	22.78 ^a^	20.75 ^bc^	19.95 ^bc^	19.31 ^c^	21.35 ^ab^	19.62 ^bc^	20.94 ^abc^	19.93 ^bc^	0.360	0.017

Note: CK, no additive treatment; BM, *B. megaterium* treatment; LB, *L. acidophilus* treatment; BMLB, *B. megaterium* and *L. acidophilus* treatment; M, molasses treatment; MBM, molasses and *B. megaterium* treatment; MLB, molasses and *L. acidophilus* treatment; MBMLB, molasses, *B. megaterium* and *L. acidophilus* treatment. DM, dry matter; CP, crude protein; NDF, neutral detergent fiber; ADF, acid detergent fiber. ^abcd^ Different superscripts mark significant differences between the treatments (*p* < 0.05).

**Table 2:** Effects of different additives on fermentation quality of rice straw haylage.

Items	Treatments								SEM	*p*-Value
	CK	BM	LB	BMLB	M	MBM	MLB	MBMLB		
pH	6.42 ^a^	6.05 ^b^	5.49 ^d^	5.41 ^d^	5.72 ^c^	5.54 ^cd^	5.56 ^cd^	5.60 ^cd^	0.402	0.032
NH_3_-N (% DM)	8.59 ^a^	8.45 ^ab^	7.70 ^ab^	5.98 ^c^	8.15 ^ab^	7.83 ^ab^	6.12 ^c^	7.56 ^b^	0.072	<0.001
Acetate (g/kg DM)	4.45 ^a^	2.19 ^c^	2.22 ^c^	1.60 ^c^	2.13 ^c^	3.83 ^a^	1.97 ^c^	2.87 ^b^	0.032	<0.001
Propionate (g/kg DM)	0.23 ^bc^	0.10 ^cd^	0.09 ^d^	0.08 ^d^	0.07 ^d^	0.23 ^b^	0.34 ^a^	0.20 ^bc^	0.013	<0.001
Butyrate (g/kg DM)	0.09 ^a^	0.06 ^b^	0.02 ^d^	0.02 ^d^	0.02 ^d^	0.04 ^c^	0.02 ^d^	0.02 ^d^	0.003	<0.001
Lactic acid (g/kg DM)	6.45 ^c^	7.74 ^bc^	11.03 ^ab^	13.1 ^a^	9.89 ^c^	10.6 ^bc^	12.5 ^a^	12.24 ^ab^	0.062	0.046

Note: CK, no additive treatment; BM, *B. megaterium* treatment; LB, *L. acidophilus* treatment; BMLB, *B. megaterium* and *L. acidophilus* treatment; M, molasses treatment; MBM, molasses and *B. megaterium* treatment; MLB, molasses and *L. acidophilus* treatment; MBMLB, molasses, *B. megaterium* and *L. acidophilus* treatment. ^abcd^ Different superscripts mark significant differences between the treatments (*p* < 0.05).

**Table 3:** Effects of different additives on lactic acid bacteria and *E. coli* counts in rice straw haylage.

Items	Treatments								SEM	*p*-Value
	CK	BM	LB	BMLB	M	MBM	MLB	MBMLB		
*Lactic acid bacteria* (lgCFU/g FM)	5.66 ^b^	6.65 ^a^	6.98 ^a^	6.88 ^a^	6.63 ^a^	6.48 ^a^	6.86 ^a^	6.90 ^a^	0.465	0.055
*E. coli* (lgCFU/g FM)	7.96 ^a^	7.39 ^ab^	7.24 ^abc^	6.28 ^c^	6.70 ^cd^	7.25 ^abc^	6.75 ^bc^	6.79 ^bc^	0.848	0.034
*Bacillus* (lgCFU/g FM)	5.25 ^d^	7.36 ^a^	6.15 ^bc^	7.28 ^a^	6.81 ^b^	7.27 ^a^	6.42 ^b^	6.67 ^b^	0.130	<0.001

Note: CK, no additive treatment; BM, *B. megaterium* treatment; LB, *L. acidophilus* treatment; BMLB, *B. megaterium* and *L. acidophilus* treatment; M, molasses treatment; MBM, molasses and *B. megaterium* treatment; MLB, molasses and *L. acidophilus* treatment; MBMLB, molasses, *B. megaterium* and *L. acidophilus* treatment. ^abcd^ Different superscripts mark significant differences between the treatments (*p* < 0.05).

**Table 4:** Effects of BMLB supplemented rice straw haylage on nutrient apparent digestibility of rice straw haylage in Hu sheep.

Items	CK	BMLB	SEM	*p*-Value
Apparent digestibility %				
DM	54.5	56.1	0.369	0.069
CP	31.9 ^b^	36.8 ^a^	0.571	0.031
NDF	60.3 ^b^	67.4 ^a^	0.822	0.027
ADF	60.1	59.6	0.598	0.151

Note: CK, no additive treatment; BMLB, Bacillus, and *L. acidophilus* treatment. DM, dry matter; CP, crude protein; NDF, neutral detergent fiber; ADF, acid detergent fiber. ^ab^ Different superscripts mark significant differences between the treatments (*p* < 0.05).

**Table 5:** Effects of BMLB treatment on ruminal fermentation characteristics in Hu sheep fed with rice straw haylage.

Items	CK	BMLB	SEM	*p*-Value
pH	7.30	7.26	0.332	0.104
VFAs				
Acetate (mmol/L)	45.4 ^a^	34.2 ^b^	2.379	0.035
Propionate (mmol/L)	5.54 ^b^	6.92 ^a^	0.077	0.042
Acetate/Propionate	8.13 ^a^	5.03 ^b^	0.398	0.015
Butyrate (mmol/L)	5.39	5.16	0.344	0.529
NH_3_-N (mg/100 mL)	10.68 ^a^	8.45 ^b^	0.250	0.047

Note: CK, no additive treatment; BMLB, *B. megaterium* and *L. acidophilus* treatment. ^ab^ Different superscripts mark significant differences between the treatments (*p* < 0.05).

**Table 6:** Effects of BMLB treatment on methane emissions from Hu sheep fed rice straw haylage.

Items	CK	BMLB	SEM	*p*-Value
Daily CH_4_ emission (mg/m^3^·d)	413.7	407.9	5.45	0.920
Daily CH_4_ emission/metabolic BW (mg/m^3^·kg·d)	26.90 ^a^	17.96 ^b^	1.840	0.041
CH_4_ emission/DMI (mg/m^3^·kg)	3.05	2.70	0.260	0.622
CH_4_ emission/NDF intake (mg/m^3^·kg)	3.81	3.17	0.319	0.367
CH_4_ emission/ADF intake (mg/m^3^·kg)	4.17	3.63	0.379	0.545

Note: CK, no additive treatment; BMLB, *B. megaterium* and *L. acidophilus* treatment. ^ab^ Different superscripts mark significant differences between the treatments (*p* < 0.05).

## Data Availability

The original contributions presented in this study are included in the article. Further inquiries can be directed to the corresponding author.
